# Cancer survivors post-chemotherapy exhibit unique proprioceptive deficits in proximal limbs

**DOI:** 10.1186/s12984-022-01010-w

**Published:** 2022-03-23

**Authors:** Allison B. Wang, Stephen N. Housley, Ann Marie Flores, Timothy C. Cope, Eric J. Perreault

**Affiliations:** 1 Department of Biomedical Engineering, Northwestern University, 355 E Erie St 21st Floor, Evanston, IL 60611 USA; 2 Department of Physical Therapy and Human Movement Sciences, Northwestern University, Chicago, IL USA; 3 Shirley Ryan AbilityLab, Chicago, IL USA; 4 School of Biological Sciences, Georgia Institute of Technology, Atlanta, GA USA; 5 Department of Medical Social Sciences, Northwestern University, Chicago, IL USA; 6 Cancer Survivorship Institute, Robert H. Lurie Comprehensive Cancer Center of Northwestern University, Chicago, IL USA; 7 W.H. Coulter Department of Biomedical Engineering, Emory University and Georgia Institute of Technology, Georgia Institute of Technology, Atlanta, GA USA; 8 Integrated Cancer Research Center, Parker H. Petit Institute for Bioengineering and Bioscience, Georgia Institute of Technology, Atlanta, GA USA; 9 Department of Physical Medicine and Rehabilitation, Northwestern, Chicago, IL USA

**Keywords:** Oxaliplatin, Proprioception, Chemotherapy-induced peripheral neuropathy, Sensorimotor, Force matching, Reaching, Postural stability, Upper-limb, Quantitative assessment

## Abstract

**Background:**

Oxaliplatin (OX) chemotherapy for colorectal cancer is associated with adverse neurotoxic effects that can contribute to long-term sensorimotor impairments in cancer survivors. It is often thought that the sensorimotor impairments are dominated by OX-induced dying-back sensory neuropathy that primarily affects the distal regions of the limb. Recent preclinical studies have identified encoding dysfunction of muscle proprioceptors as an alternative mechanism. Unlike the dying-back sensory neuropathy affecting distal limbs, dysfunction of muscle proprioceptors could have more widespread effects. Most investigations of chemotherapy-induced sensorimotor impairments have considered only the effects of distal changes in sensory processing; none have evaluated proximal changes or their influence on function. Our study fills this gap by evaluating the functional use of proprioception in the shoulder and elbow joints of cancer survivors post OX chemotherapy. We implemented three multidirectional sensorimotor tasks: force matching, target reaching, and postural stability tasks to evaluate various aspects of proprioception and their use. Force and kinematic data of the sensorimotor tasks were collected in 13 cancer survivors treated with OX and 13 age-matched healthy controls.

**Results:**

Cancer survivors exhibited less accuracy and precision than an age-matched control group when they had to rely only on proprioceptive information to match force, even for forces that required only torques about the shoulder. There were also small differences in the ability to maintain arm posture but no significant differences in reaching. The force deficits in cancer survivors were significantly correlated with self-reported motor dysfunction.

**Conclusions:**

These results suggest that cancer survivors post OX chemotherapy exhibit proximal proprioceptive deficits, and that the deficits in producing accurate and precise forces are larger than those for producing unloaded movements. Current clinical assessments of chemotherapy-related sensorimotor dysfunction are largely limited to distal symptoms. Our study suggests that we also need to consider changes in proximal function. Force matching tasks similar to those used here could provide a clinically meaningful approach to quantifying OX-related movement dysfunction during and after chemotherapy.

## Introduction

Oxaliplatin (OX) is effective as the primary treatment for metastasized colorectal cancer and an adjuvant therapy for other gastrointestinal neoplasms [1–4]. Despite its efficacy, accumulating OX doses can result in adverse neurotoxic effects, leading to movement impairments, such as a loss of dexterity, postural imbalance, and falls [5, 6]. The dysfunction resulting from OX neurotoxicity might require physicians to reduce the dose, delay treatment, or even terminate treatment prematurely, all of which compromise treatment effectiveness [1, 7]. This dysfunction can persist in a substantial number of patients for months and years after treatment completion, negatively impacting quality of life [7–9]. With the estimated number of cancer survivors of colorectal cancer increasing to 2 million over the next decade, monitoring and treating OX-induced impairments has become an urgent issue. However, this effort is hindered by an inadequate understanding of OX-induced movement impairments.

Descriptions of OX-induced movement impairments have come largely from surveys of symptoms and functional limitations, with few good quantitative measures [10]. Many of the commonly used clinical surveys focus on monitoring the severity of distal sensory symptoms, such as numbness, tingling, burning, pain, and loss of light touch, as well as functional difficulty associated with these symptoms such as using utensils, dressing, walking, and writing. These surveys are easy to use and can communicate the severity of neurotoxicity to health care providers, but they provide little insight into the etiology of movement impairments. Many survey questions focus on sensory symptoms in hands/fingers and feet/toes, mainly because dying-back sensory neuropathy is commonly considered as the primary mechanism underlying OX-induced neurotoxicity and movement impairments. Dying-back sensory neuropathy is a progressive degeneration of large fiber sensory neurons resulting from OX accumulation. It is marked by reduced compound sensory nerve action potential amplitudes and sensory paresthesia distributed in a ‘stocking and glove’ pattern. It is commonly thought that dying-back sensory neuropathy contributes to movement impairments because sensory feedback in hands and feet is essential for guiding our interactions with the external environment. A few quantitative studies established associations between sensory symptoms and measures of balance and gait [10] but they often involved cancer survivors with mixed neurotoxic chemotherapy agents and did not investigate factors beyond dying-back sensory neuropathy. Evidence suggests that some cancer survivors could present movement impairments without dying-back neuropathy [5]. Thus, monitoring for dying-back sensory neuropathy might not be sufficient to understand OX-induced movement impairments fully.

Recent preclinical research has identified encoding dysfunction of muscle proprioceptors as an alternative mechanism that may contribute to OX-induced movement impairments [11–13]. These studies have demonstrated that OX alters the proprioceptive information received by the spinal cord. Specifically, sensory neurons originating within muscle proprioceptors consistently failed to encode features of muscle length and force. For example, it was observed that sensory neurons from both muscle spindles and Golgi tendon organs failed to maintain firing in response to a sustained muscle stretc.h in rats treated with OX [11, 13]. Several lines of evidence suggest that this encoding dysfunction is linked to OX-induced ion channelopathy within the sensory neurons, although the exact biophysical mechanism and the extent of this dysfunction in other neurons remains to be clarified [11–13]. Regardless of the mechanisms, encoding dysfunction can occur in the absence of dying-back sensory neuropathy and can contribute to substantial movement dysfunction in preclinical models of chemotherapy [11–13].

Muscle proprioceptors encode various muscular kinematic and kinetic features that are critical for perceiving limb kinesthesia and muscular force, coordinating movements, and guiding our interactions with the external environment. Importantly, these receptors are distributed throughout the muscles of our body. Thus, unlike dying-back sensory neuropathy that primarily alters distal sensory feedback, encoding dysfunction of proprioceptors will likely impair proprioception in both proximal and distal joints. Previously, generalized loss of proprioception has been associated with an inability to coordinate multidirectional and multi-segmental movements and to maintain steady postures and motor output [14–16], underscoring the functional consequence of proprioceptive dysfunction. The preclinical studies provide strong evidence for OX-induced widespread proprioceptive deficits, but no studies have investigated proprioceptive dysfunction in the proximal joints and its link to movement impairments in human cancer survivors. In-depth quantification of proprioception dysfunction and its functional consequences could provide insight into the mechanisms underlying the OX-induced movement impairments and indicate more discriminating ways of monitoring and treating dysfunction.

This study aims to determine if proximal proprioceptive dysfunction contributes to movement dysfunction in cancer survivors after OX chemotherapy. We quantified the use of proprioception in controlling upper limb position, force, and posture using reaching, isometric force matching, and postural stability tasks, respectively. Each task consisted of 6 directions to evaluate the spatial consequence of proprioceptive dysfunction in multi-segment control. We fixed the dominant hand and wrist in an orthosis to emphasize elbow and shoulder use, as these joints are proximal and less likely to have sensory neuropathy. All subjects completed tasks with and without visual feedback to differentiate contributions from proprioception and vision. We hypothesized that cancer survivors after OX chemotherapy would have impaired task performance linked to proximal proprioceptive dysfunction. Our results have implications for guiding the development of targeted assessments and interventions for OX-related movement dysfunction.

## Methods

### Participants

The participants of this study included 13 cancer survivors who had completed their oxaliplatin-containing chemotherapy and had not received any other neurotoxic agents and 13 healthy controls who were cancer-free and chemotherapy naïve (Table 1). Participants were included in the study if they were 18 years of age or older, could understand task instruction, and did not have any diagnoses of sensory disorders (e.g., Guillain-Barre syndrome, B12 sensory neuropathy), central nervous system disorders (e.g., spinal cord injury, brain injury, multiple sclerosis, Parkinson’s disease), other systemic medical conditions (e.g., fibromyalgia, rheumatoid arthritis, diabetes), or upper limb injuries. All participants provided written informed consent before data collection. The scientific review committee of Robert H. Lurie Comprehensive Cancer Center and the Institutional Review Board of Northwestern University approved this study.

### Assessment of signs, symptoms, and quality of life related to chemotherapy-induced peripheral neuropathy (CIPN)

Recommended clinical measures, including the European Organization for Research and Treatment of Cancer Quality of Life (EORTC QLQ) CIPN20 and C30 questionnaires, the modified Total Neuropathy Score (mTNS), and a visual analog pain scale, were used to assess OX-related signs and symptoms and quality of life [17].

The EORTC QLQ-CIPN20 questionnaire is a validated instrument that assesses sensory, motor, and autonomic symptoms and functional limitations related to CIPN. It consists of 20 items, and each item is scored from 1 (not at all) to 4 (very much) based on the severity of symptoms experienced by patients. The sensory, motor, and autonomic subscale scores were linearly transformed to a 0–100 scale, with higher scores indicating more severe symptoms [18].

Version 3.0 of the EORTC QLQ-C30 questionnaire is a well-validated and widely used questionnaire designed to assess the impact of cancer and its treatments on the core set of quality of life issues. It consists of 30 items that cover six functional domains (physical, role, emotional, cognitive, social, and global health status) and nine symptoms (fatigue, nausea/vomiting, pain, dyspnea, sleep disturbance, appetite loss, constipation, diarrhea, and financial impact). Except for two global health items that are rated from 1 (very poor) to 7 (excellent), all other items are rated from 1 (not at all) to 4 (very much). Scores from the C30 were linearly transformed to a 0–100 scale, with a higher score indicating worse function and quality of life [18].

The mTNS is a validated measure that consists of both subjective and objective items designed to assess the severity of CIPN. The subjective items ask participants to rate the severity of the sensory, motor, and autonomic symptoms. A licensed physical therapist administered the objective items to test for the presence and severity of the deficits in pinprick sensitivity, vibration sensitivity, muscle strength, and deep tendon reflexes. All items were rated on a 4-point scale. The total score was linearly transformed to a 0–100 scale with a higher score corresponding to worse symptoms [18].

### Proprioception-focused sensorimotor assessment

Three multidirectional sensorimotor tasks, target reaching, force matching, and postural stability, were used to assess the use of proprioceptive information. These sensorimotor tasks, which rely on the kinesthetic and force components of proprioception, were adapted from previous experimental paradigms used to investigate the functional consequence of proprioceptive deficits [14–16, 19, 20]. Similar paradigms have also been used in recent studies to evaluate proprioceptive function in healthy individuals and patients with stroke [21–23].

#### Equipment and setup

**Fig. 1:**
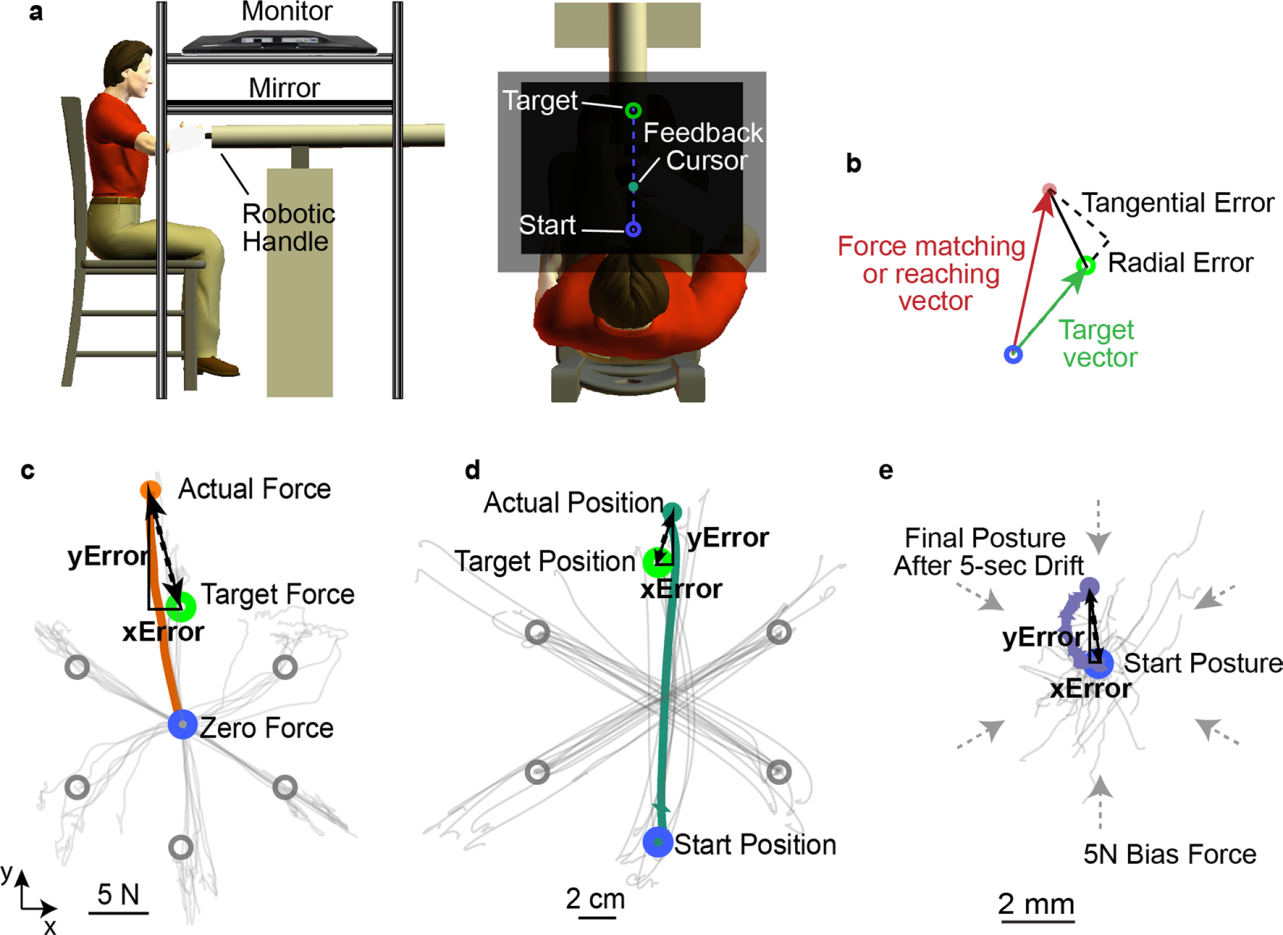
Experimental setup. **a** side and top views of subject position during data collection. **b** definitions of radial and tangential errors for the force matching and target reaching tasks. **c**–**e** are three sensorimotor tasks tested. Each thin trace is a trial. **c** Force matching: Subject generates a 10 N force vector against the robotic handle in locked mode, first with the feedback cursor visible, and then subject generates the same force vector with the feedback cursor invisible. The feedback cursor represents the force vector. **d** Target reaching: The subject moves the robotic handle from a start position to a target position. The feedback cursor representing hand position is visible when there is visual feedback and invisible when there is no visual feedback. **e** Postural stability: The subject stays within a start position for 5 s while resisting a 5 N-force vector from the robot. The feedback cursor represents the hand position, and the visibility is randomly assigned to be visible or invisible

A three-degree-of-freedom robotic manipulator (Haptic Master; Moog SCS, Nieuw-Vennep, The Netherlands) was used to implement the sensorimotor tasks and record kinematics and force data (Fig. 1). Details of the equipment have been provided previously [24]. Participants sat in a Biodex chair (Biodex Medical Systems, Shirley, NY) with the trunk secured. The hand and wrist were constrained and securely attached to the robot using a custom-fitted plastic orthosis mounted to a gimbal at the end of the manipulator. The orthosis fixed the hand and wrist in a neutral position and extended approximately one-third of the distance from the wrist to the elbow so that the generated forces and motions resulted primarily from actions at the elbow and shoulder. The tested arm was supported against gravity by a passive multilink device (Jaeco, Hot Spring, AR) to avoid fatigue. In the target reaching and postural stability tasks, the robot was used in an admittance control mode so participants could move their arms freely in the horizontal plane. The start position, target position, and hand location were displayed on a monitor covering the arm. A similar arrangement was used in the force matching task except that the robot was used in an isometric mode, and the display showed the voluntary force exerted by the subject on the robot rather than hand position. The feedback cursor of the hand location or force can be switched on and off to emphasize vision or proprioception use. All participants completed a practice session to get familiar with the tasks and conditions. These practice data were not used in subsequent analyses. Participants completed the three tasks in random orders.

##### Target reaching

Six positions separated by 15 cm in the workspace across the mid-chest area were used. Only two positions were shown in a trial – a home position and a target position. Participants initiated the trial by moving the hand cursor into the home position. After participants maintained the hand cursor in the home position for 0.5 s, a visual cue (hand cursor turns from red to green) and an audio sound signaled the participants to reach the target. Participants were instructed to use a self-selected reaching speed and maintain the hand cursor in the target until the end of the trial. In trials with visual feedback, the hand cursor was visible for the entire reach; in trials without visual feedback, the hand cursor became invisible after participants initiated the reach. The visible and invisible trials were separately tested in 4 blocks, and the orders of the blocks were randomized. Each reaching trial lasted 7 s, and each direction was repeated 6 times.

##### Force matching

The Haptic Master robot was set to the isometric mode, preventing the participant’s tested arm from moving freely. Each participant’s hand was set to a position ~ 20 cm across their mid-chest. Six force vectors of 10 N were used. Participants initiated the trial by relaxing their arm (staying in zero force) for 0.5 s. Then the force feedback cursor turned green, and an audio sound signaled the participants to generate a force that matches the target force vector with visual feedback. Participants were instructed to maintain the force in the target, memorize the force vector, and relax when the target disappears (7 s after initiating the trial). After 3 s, the target reappeared, and participants were instructed to regenerate the remembered force without visual feedback and maintain the force until the trial ended (7 s after the target reappears). Seven seconds was selected to allow sufficient time for participants to generate and maintain a target force, but not too long to cause fatigue. Each force vector was repeated 6 times and completed in 3 testing blocks. Participants took rest breaks in between blocks.

##### Postural stability

Participants maintained a static posture with their hand resting ~ 20 cm across their mid-chest while resisting a bias force vector. Participants initiated the trial by entering the home position. Then the HapticMaster robot applied a bias force vector of 5 N that would perturb the participants. Participants were instructed to maintain their hand cursor in the home position while resisting the force. After they stayed in the home position for 3 s, the hand cursor’s visibility would be switched off during invisible trials and kept visible during visible trials. Participants would continue to hold the bias force until the trial ended after 5 s. The orders of visible and invisible trials were randomized. Six bias force vectors were used, and each was repeated 6 times and completed in 3 testing blocks. Participants took rest breaks in between blocks.

We chose these sensorimotor tasks over position matching tasks used in other studies [25–27] because they incorporate both the kinesthetic and force aspects of the proprioception. Although kinesthetic and force components of proprioception are both needed to complete all tasks, target reaching relies more on limb kinesthesia whereas force matching relies more on a sense of muscular force; the postural stability task requires information about both kinesthesia and force. It is important to note that all tasks include sensory and motor components, as is relevant to most functional tasks dependent on proprioception. All tasks were evaluated in six directions within a horizontal workspace to generate a range of muscle activations sensory inputs relevant to a variety of daily tasks. Furthermore, multidirectional tasks require coordination between muscles crossing the elbow and shoulder, and compensation for the anisotropic biomechanical properties of the human arm [28, 29]. Thus, they are likely harder to complete if there are proprioceptive deficits.

##### Data analysis

**Fig. 2:**
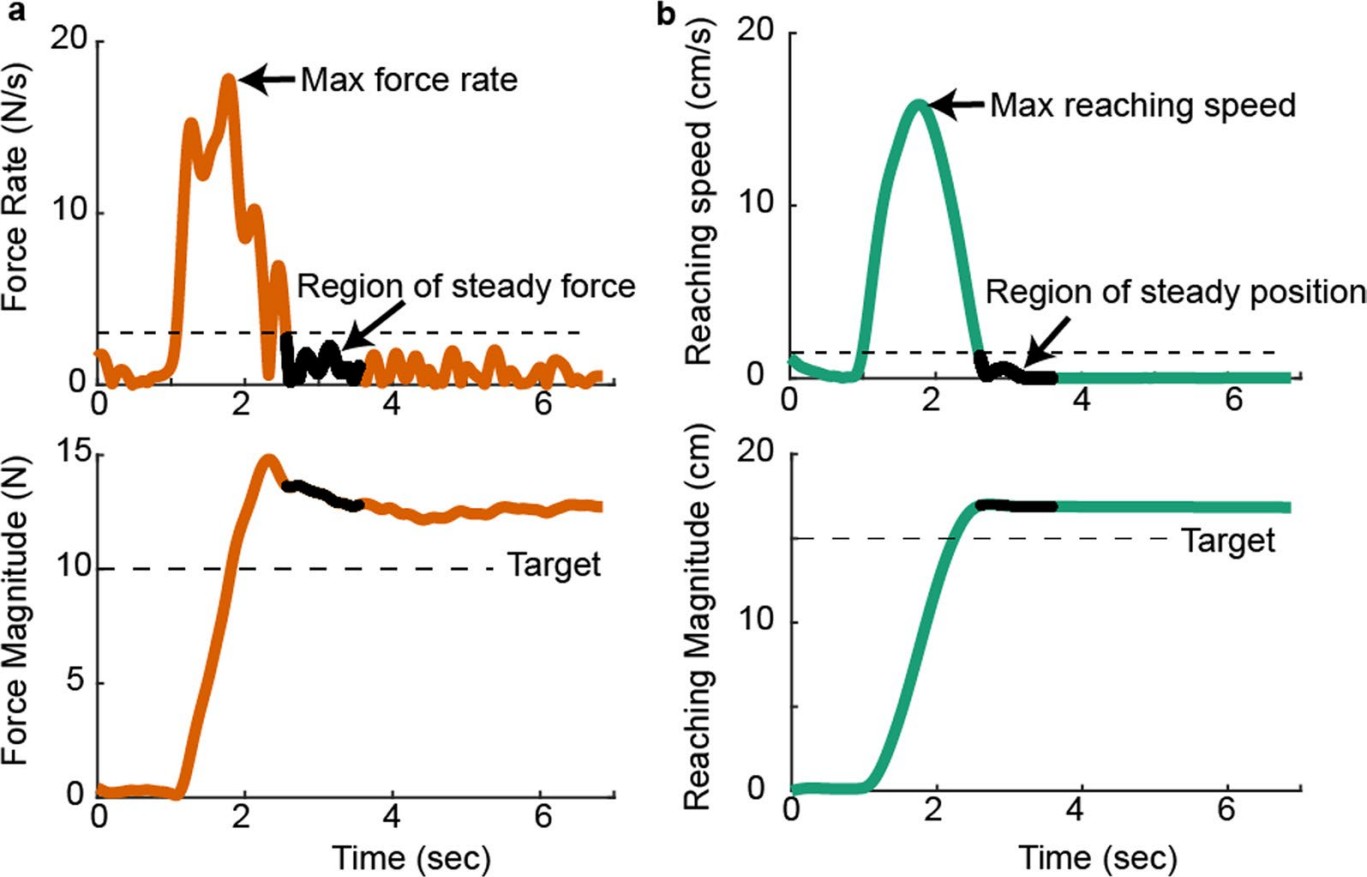
Method for computing steady-state force during the force matching task (**a**) and steady-state position during the target reaching task (**b**). 8% of the maximal reaching speed and 15% of the maximal force rate were used to determine the steady-state of the reaching task and force-matching task, respectively. Averaged force and position within the region of steady position were used to calculate performance errors

Kinematic and force data of the sensorimotor tasks were sampled at 2 kHz and were smoothed using a 4th order, zero-lag, low-pass Butterworth filter with a cut-off frequency of 8 Hz. Given that participants were allotted 7 s to reach a target and match a force, the steady-state of the hand position and force were first identified for the target reaching and force matching tasks, respectively (Fig. 2). The steady hand position was defined as when the rate of position change was maintained below 8% of the maximal reaching speed for 1 s. The steady force was defined as when the rate of force change was maintained below 15% of the maximal force rate for 1 s. For trials with multiple steady states, the earliest one was used. Trials were discarded if the participants could not maintain a steady position or force for 1 s (7.7% of the trials). The data in the steady period were averaged and used to evaluate task performance. We evaluated the accuracy and precision of the performance. Performance accuracy quantifies the x and y errors relative to the target. Given that six targets were used for the force matching and target reaching tasks, we also quantified the radial and tangential errors relative to the target vector (Fig. 1b) to allow reasonable comparisons across different target directions for the two tasks. Performance precision quantified the spread of the data around the mean performance for the same task condition. The standard deviation of the distance of each point from the mean center was used to quantify the spread.

### Statistical analysis

Our central hypothesis was that cancer survivors have impaired use of proprioceptive information in sensorimotor tasks compared to healthy controls. We compared the accuracy and precision of the sensorimotor performance between cancer survivors and controls using mixed-effect models. For performance accuracy, we implemented multivariate and multilevel mixed-effect models. We set the participant group (cancer survivors vs. controls), visual feedback condition (on vs. off), and direction as fixed effects, and subject as a random effect. Task accuracy, measured by the radial and tangential errors in the force matching and target reaching tasks, and x and y errors in the postural stability task, was set as the dependent variable. Separate analyses were performed for each task. For performance precision, we implemented a univariate mixed-effect model. We set the participant group, visual feedback condition, and direction as fixed effects, subject as a random effect, and task precision as the dependent variable. Separate analyses were performed for each task. All trials were considered in the analysis to account for the variability within each subject appropriately. Linear hypothesis tests on the fixed effects were performed using F-tests (implemented by the coefTest function in MATLAB) during the post-hoc analyses. We expected the effects of visual feedback condition and participant group to be significant. Significant interactions between the two factors would indicate that cancer survivors weighted the visual system differently during sensorimotor tasks, consistent with changes in proprioception use.

Lastly, to investigate if the sensorimotor deficits in cancer survivors were related to clinical signs and symptoms of CIPN, we completed a Pearson correlation analysis. We computed the subject mean of performance accuracy and precision for each sensorimotor task and correlated the proprioception-related changes in these parameters to the score of CIPN20, C30, and TNSc, and their sub-scores. We were specifically interested in the sensory and motor sub-scores of CIPN20 as sensory and motor symptoms are common, and these sub-scores might be more relevant to the sensorimotor function.

All statistical analyses were performed in MATLAB (2020b, Mathworks, Natick, MA). Significance was evaluated against a p-value of 0.05.

## Results

### CIPN signs and symptoms

**Fig. 3:**
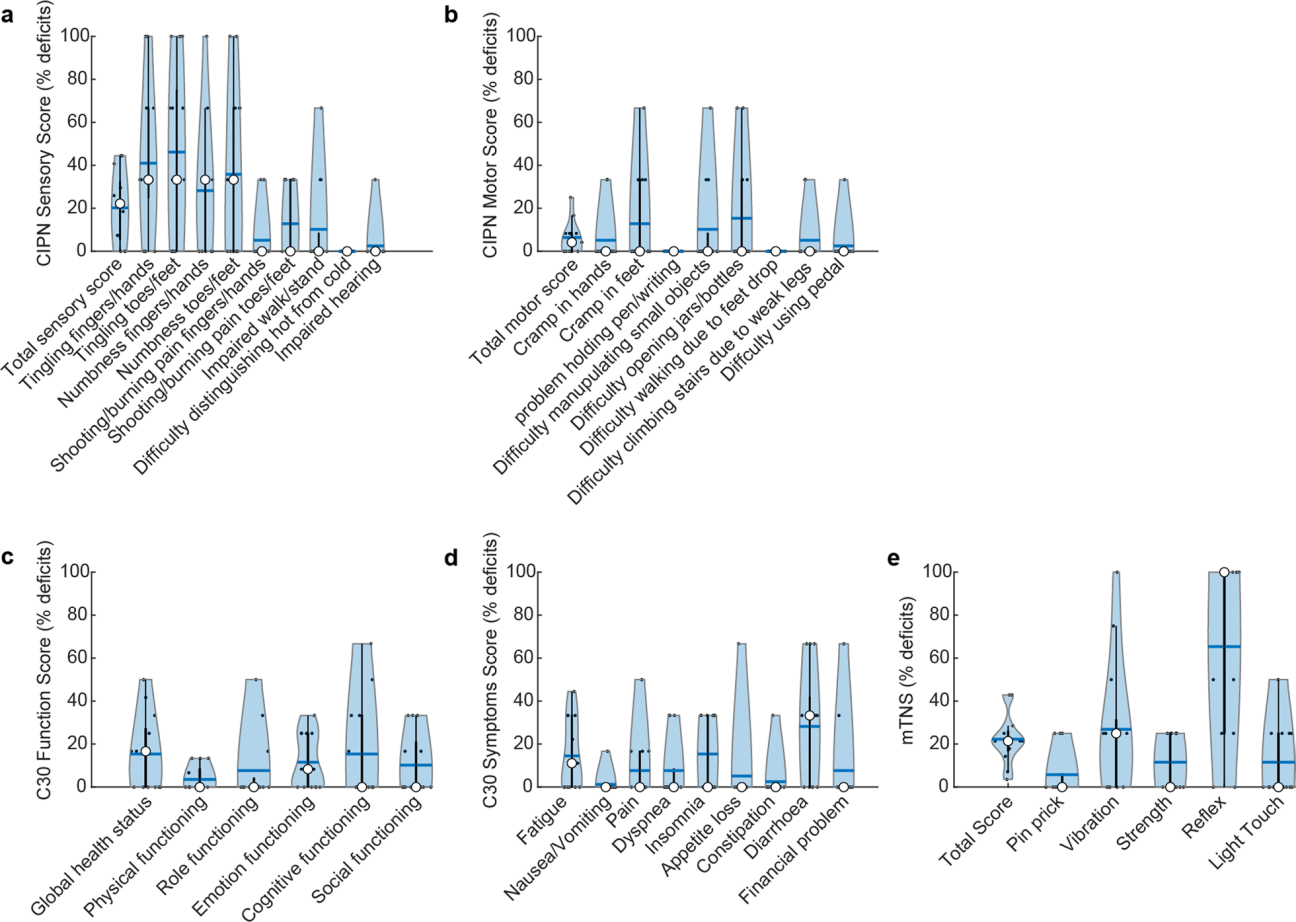
Summary of the sensory, motor, and functional deficits based on the CIPN 20 questionnaire (**a**, **b**), C30 questionnaire (**c**, **d**), and mTNS (**e**). Data are group results of cancer survivors. The white circle is the median, the black dot is an individual data point, the horizontal blue bar is the mean, and the shaded area is the kernel density estimate of the data

All cancer survivors reported some degree of decreased quality of life as well as symptoms commonly associated with CIPN (Fig. 3). Commonly reported categories for decreased quality of life were emotional function, global health, diarrhea, insomnia, and fatigue. The most common sensory symptoms were numbness and tingling in the hands and feet, and the most reported motor impairments were difficulty opening jars and cramps in the feet. Sensory symptoms were slightly more severe and common than motor symptoms. 77% of cancer survivors reported at least one sensory symptom, whereas 62% reported at least one motor symptom. The average sensory deficit score was 20.2%, whereas the motor deficit score was 6.4%. All cancer survivors except one had impaired or absent ankle or knee reflexes with mixed deficits in light touch sensitivity, pinprick sensitivity, vibration sensitivity, and strength. All healthy controls had no apparent deficits in light touch sensitivity, pinprick sensitivity, vibration sensitivity, strength, and reflexes. Additionally, healthy controls reported no deficits in the CIPN20 questionnaire, except for one subject who reported difficulty in hearing. They also reported a mildly decreased quality of life rating in global health, emotional function, cognitive function, fatigue, pain, and insomnia.

### Task accuracy

**Fig. 4:**
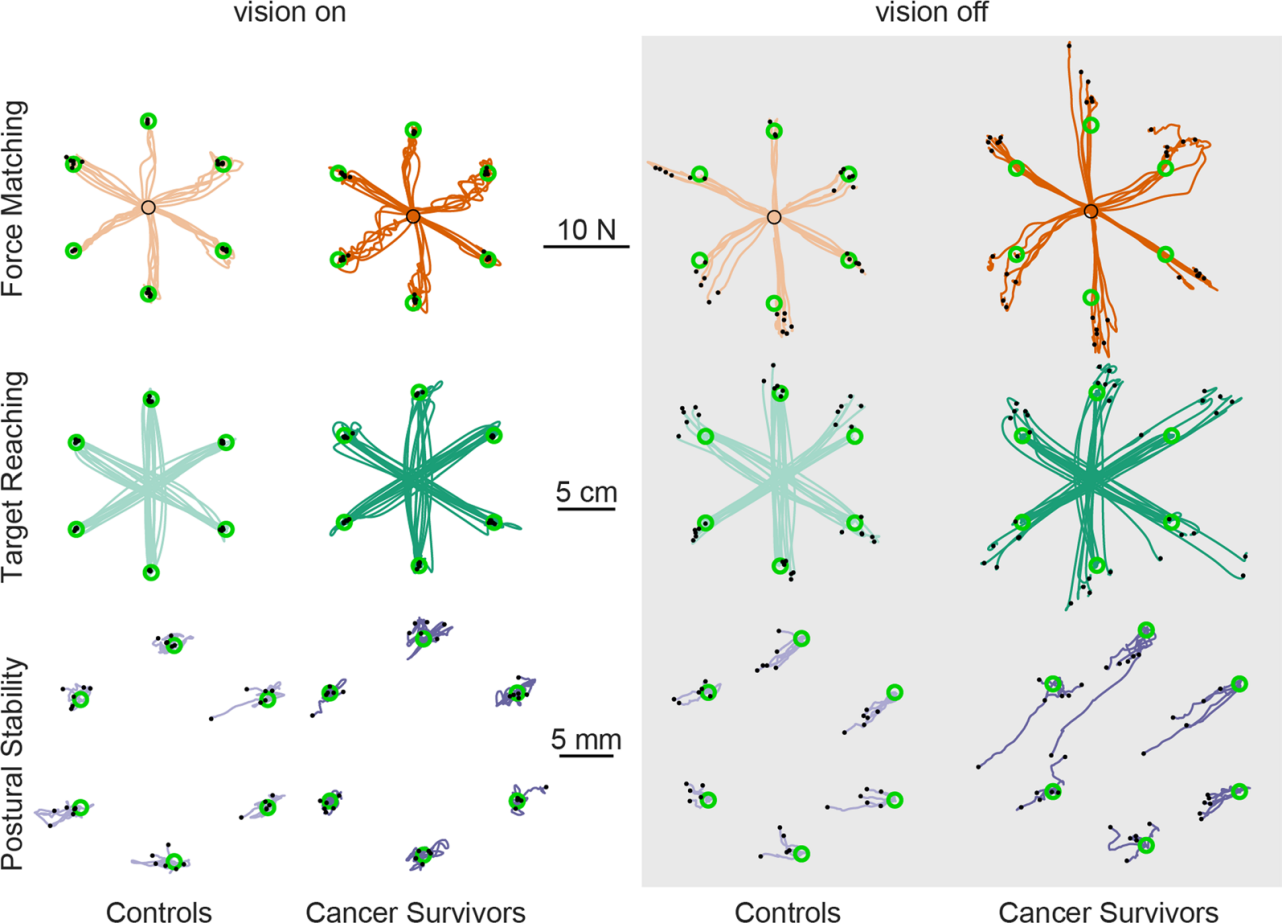
Sample data for a representative control subject (lighter traces) and a cancer survivor (darker traces). Each trace is from a single trial. Solid black dots at the end of the traces represent the end of the trial and were used to calculate performance errors relative to targets (green open circles). In the postural stability task, the performance traces were separated by the direction of the bias force

**Fig. 5:**
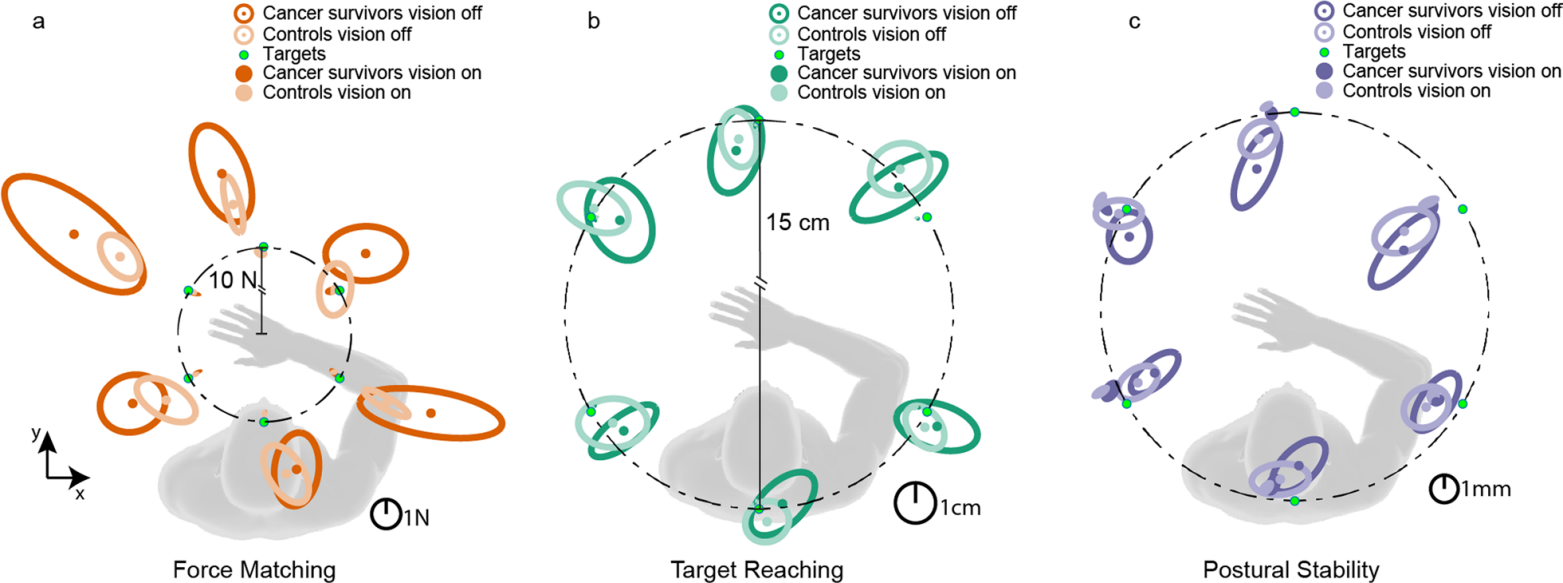
Differences in performance accuracy between cancer survivors (darker color trace) and controls (lighter color trace). Data are group results. Darker and lighter dots are the mean performance with respect to targets (green). Ellipse indicates 95% confidence intervals of the mean estimate. Averaging across directions, the proprioception-related accuracy was significantly greater in cancer survivors for the force matching task (*p* = 0.003), but not for the target reaching (*p* = 0.42) and postural stability (*p* = 0.45) tasks

All participants completed the three sensorimotor tasks with no reports of significant difficulty. Typical performance can be seen by the data from a representative control subject and cancer survivor (Fig. 4). When visual feedback was provided, both participants were comparably accurate in all tasks. The accuracy of both participants deteriorated when visual feedback was removed, but the cancer survivor appeared to present with larger errors across all three tasks. For instance, in the force matching task, both individuals overshot the targets without visual feedback, but the cancer survivor overshot the targets more. Most of these observations held in the group results (Fig. 5).

When visual feedback was provided, we found no significant differences in accuracy between the groups of cancer survivors and healthy controls for the force matching (Fig. 5a, Mean Difference (95% lower and upper bound): 0.03 (-0.05, 0.13) N, *p =* 0.67) and target reaching tasks (Fig. 5b, 0.12 (− 0.078, 0.54) mm, *p =* 0.60) across all target directions. Interestingly, there was a small but statistically significant increase in postural drift in healthy controls compared to cancer survivors for the postural task (Fig.  5c, 0.22 (0.075, 0.37) mm, *p =* 0.002).

When visual feedback was removed, the cancer survivors exhibited larger errors than the control group in both the force matching and postural stability tasks. Both groups consistently overshot the targets in the force matching task, though cancer survivors had larger errors (Fig. 5a, 1.5 (0.31, 1.98) N, *p =* 0.005). Cancer survivors also had a significantly different postural drift pattern compared to controls in the postural stability task (Fig. 5c, 0.41 (0.038, 0.84) mm, *p =* 0.03). The difference in target reaching accuracy did not reach a significant threshold (Fig. 5b, 0.84 (− 3.2, 5.6) mm, *p =* 0.42).

The change in accuracy from vision to no vision, which we refer to as proprioception-related accuracy, was significantly greater for the cancer survivors relative to the healthy controls only in the force matching task (1.18 (0.36, 2) N, *p =* 0.003), not in the postural stability task (0.20 (− 0.18, 0.66) mm, *p =* 0.45), or in the targeting reaching task (0.73 (− 3.5, 5.5) mm, *p =* 0.42). This suggests that cancer survivors relied more on visual feedback than controls when completing the force matching task. Since the proprioception-related accuracy is more relevant to proprioceptive changes, we compared the effect of direction on proprioception-related accuracy between the groups.

The difference in proprioception-related accuracy between the two groups did not change significantly with the direction of the relevant motions or forces across all tasks (all *p* > 0.05/6). However, there was substantially more variability in the estimated mean responses for the cancer survivors than the control group in the force matching task, and this variability changed with direction, as can be observed by the confidence intervals in Fig. 5a. This increased variability could arise from low trial-to-trial repeatability (precision) within subjects or differences in performance across subjects. We, therefore, compared the task precision and inter-subject variability to assess these possibilities.

### Task precision

**Fig. 6:**
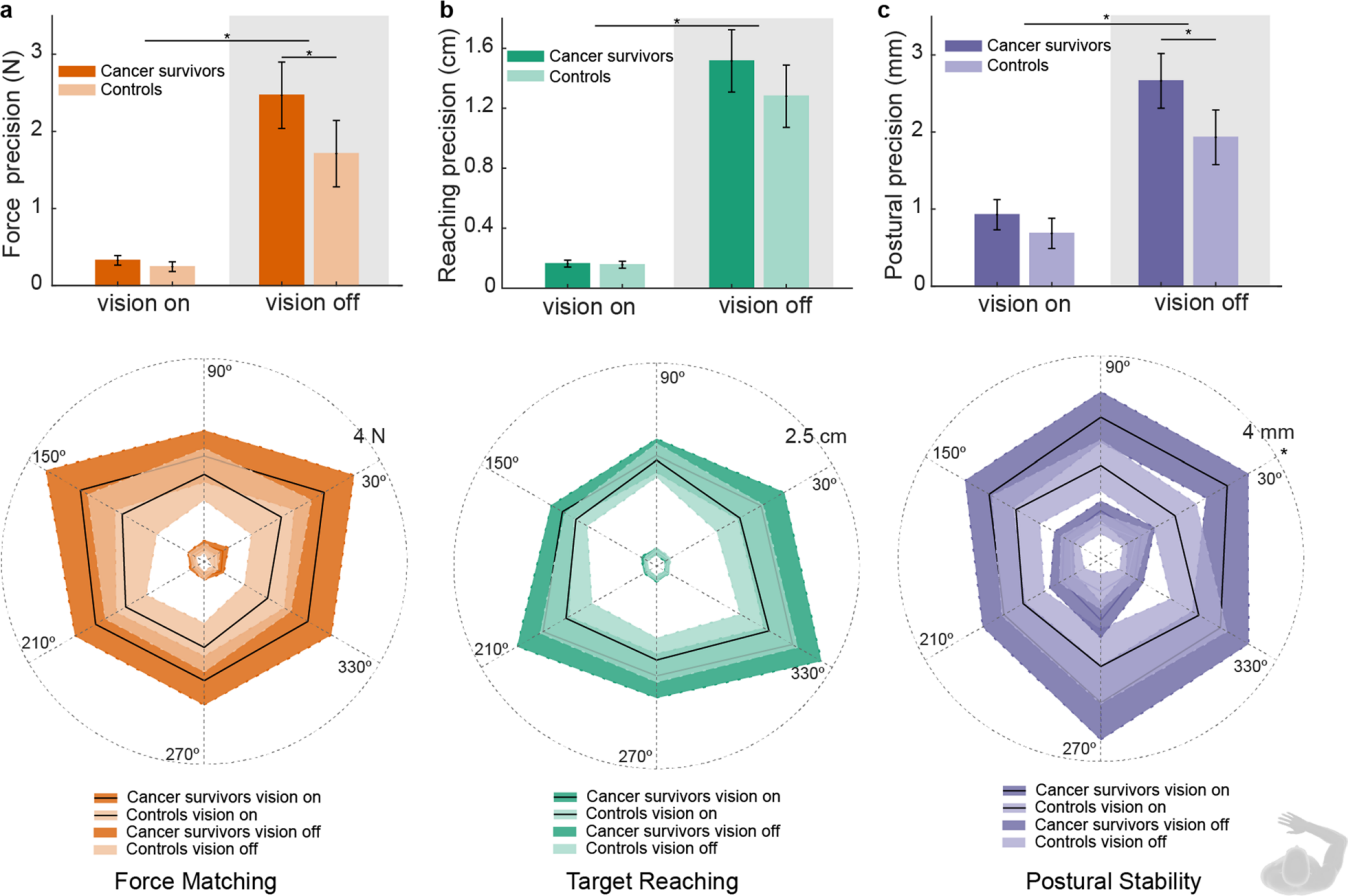
Differences in performance precision between cancer survivors (darker color trace) and controls (lighter color trace). Data are group results. Precision is defined as the standard deviation of the distance between the measure in each task (force or position) and the target given to the subject. Top panels: **a**–**c** Bar indicates mean performance precision, and error bars are 95% confidence intervals around the mean. Asterisk indicates groups with significant differences. Bottom panels of **a**–**c**: same data as in the top panel but separated by directions

With vision present, cancer survivors tended to have decreased precision in the force matching and postural control tasks compared to controls, though these differences did not reach statistical significance when averaging across all directions (Fig. 6a, Force matching: 0.082 (− 0.046, 0.13) N, *p =* 0.066; Fig. 6b, Postural stability: 0.24 (− 0.14, 0.41) mm, *p =* 0.082). In contrast, the precision of the target reaching task was more consistent across groups when vision was present (Fig. 6c and 0.072 (− 0.26, 0.40) mm, *p =* 0.66).

Precision decreased for all subjects in the absence of vision. We refer the change in precision from vision to no vision as proprioception-related precision. The average proprioception-related precision across directions was larger in the cancer survivors than in the healthy controls for the force matching (0.67 (0.07, 1.28) N, *p =* 0.030) and postural stability (0.49 (0.010, 0.097) mm, *p =* 0.049) tasks. It did not reach statistical significance in the target reaching task (2.3 (− 0.54, 5.1) mm, *p =* 0.11). Across all tasks, the difference in proprioception-related precision between the two groups did not change significantly with direction after correcting for multiple comparisons (all *p* > 0.05/6). The most significant difference was observed for the postural stability task in the direction of 30° (1.1 (0.33, 1.8) mm, *p =* 0.007).

### Correlation of sensorimotor performance with CIPN signs, symptoms, and functional limitations

**Fig. 7:**
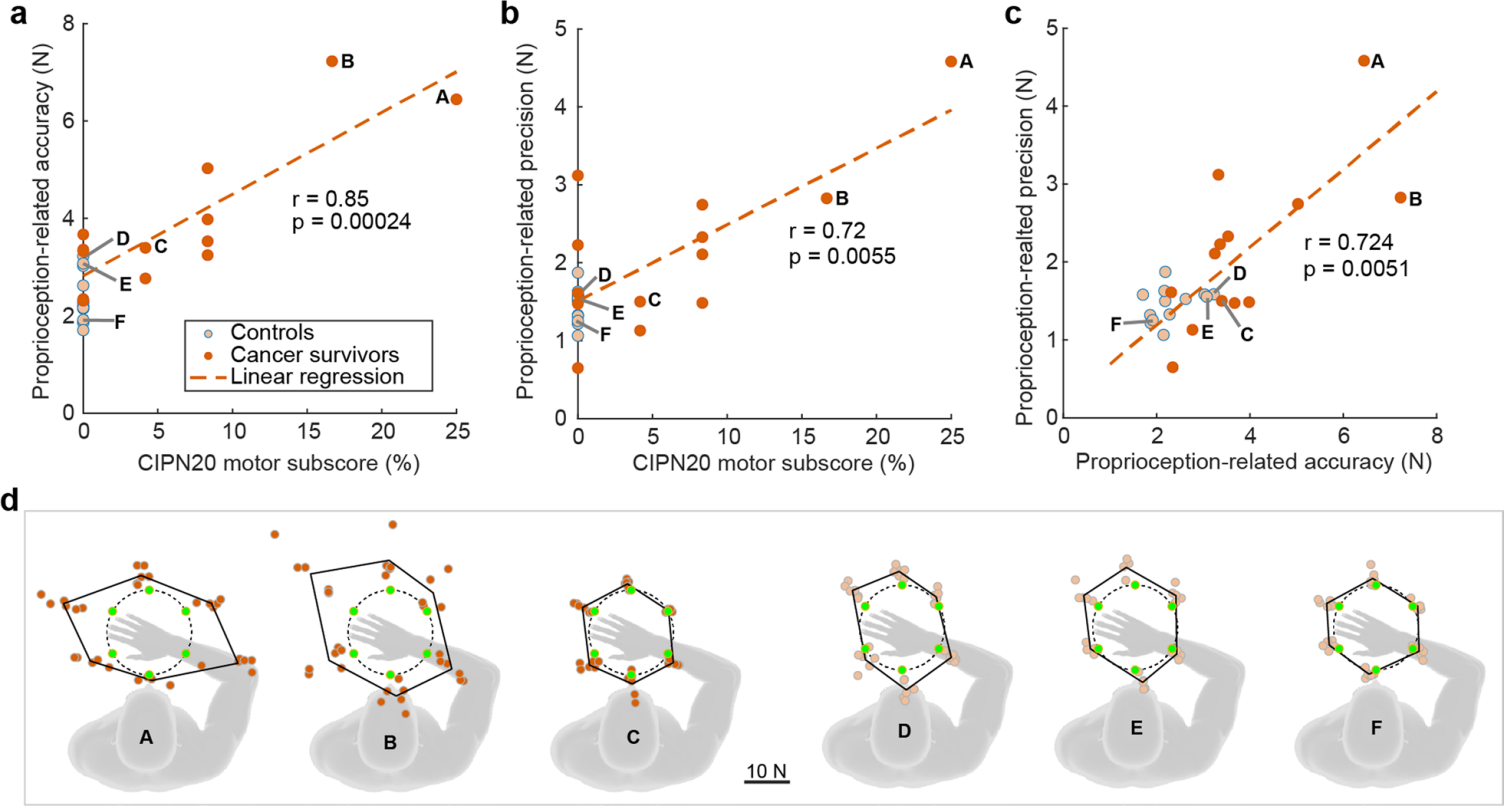
The average proprioception-related accuracy (**a)** and proprioception-related precision (**b)** were correlated with patient-reported deficits in the CIPN20 motor sub-score. **c** The average proprioception-related accuracy and prevision were correlated with each other. Each dot represents an individual subject. **d** Force matching performance of cancer survivors **A**–**C** and controls (**D–F**). A and B are cancer survivors with worse performance, and **D** and **E** are controls with worse performance. **C** and **F** are averaged cancer survivors and controls, respectively. Green dots are targets. Black lines connect the mean performance in each direction. Plotted subjects were labeled in (**a**–**c**)

Across the three tasks, cancer survivors presented with the largest deficits in the force matching task, and these deficits were significantly correlated with their perceived functional limitations. We found that the correlations between the CIPN20 motor sub-score and proprioception-related accuracy (Fig. 7a, r = 0.861, *p* < 0.001) and between CIPN20 motor sub-score and proprioception-related precision (Fig. 7b, r = 0.718, *p =* 0.006) were significant in cancer survivors. The same cancer survivors who demonstrated poor task accuracy also tended to have poor task precision (Fig. 7c), suggesting that either metric could be used as a measure of movement impairment. Interestingly, although our cancer survivors reported sensory deficits more frequently than motor deficits (Fig. 3), the correlation between our measures of force matching performance and the CIPN20 sensory sub-score did not reach significance (accuracy: *p =* 0.11; precision: *p =* 0.29). Correlations between all clinical scores (CIPN20, C30, and mTNS) and the performance measures for target reaching and postural stability were also not significant.

**Fig. 8:**
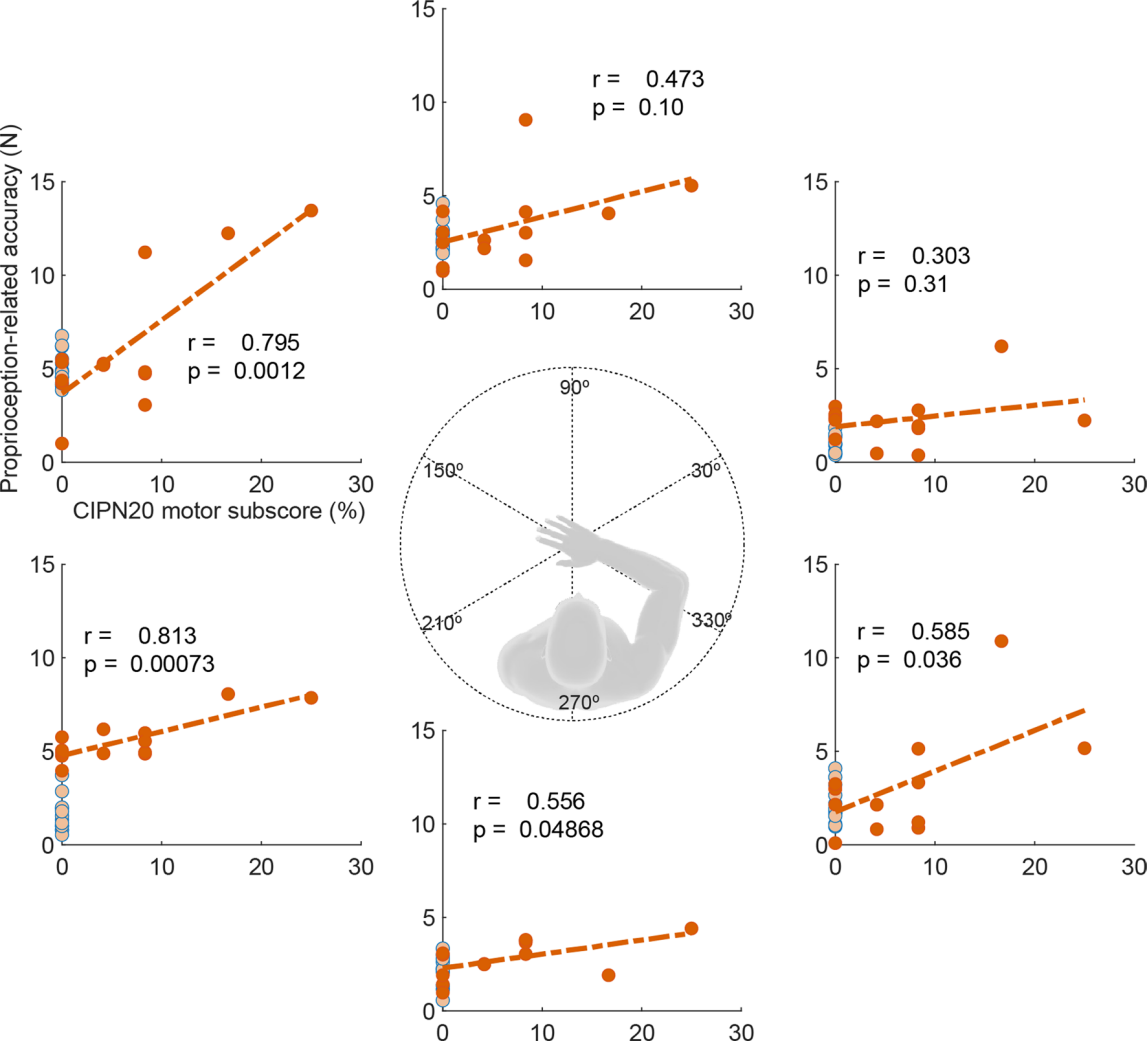
The proprioception-related accuracy at each direction was correlated with patient-reported deficits in CIPN20 motor sub-score. Each dot represents an individual subject

The force matching task could be a useful measure of movement impairment, but the task required more than 30 min to complete, limiting its clinical application. To determine if a simplified version is appropriate for assessing movement dysfunction associated with chemotherapy, we correlated the CIPN20 motor sub-score with proprioception-related accuracy in each force direction (Fig. 8). We found that the most significant correlations were in the directions of 150° (r = 0.8, *p =* 0.001), 210° (r = 0.81, *p* < 0.001), and 330° (r = 0.585, *p =* 0.036). The correlations with precision were also significant in these three directions (150°: r = 0.79, *p =* 0.001; 210°: r = 0.87, *p =* 0.001; 330°: r = 0.694, *p =* 0.012). These results suggest that force matching in one of the three directions could be useful as a simplified version for assessing motor impairment associated with chemotherapy, making the clinical translation of the task more feasible.

In contrast to the CIPN20 motor sub-scores, force matching performance was not correlated with chemotherapy treatment duration (r = 0.17, *p* = 0.58), body weight normalized cumulative dosage (r = 0.2, *p* = 0.5), or cumulative dosage (r = 0.09, *p* = 0.78), which have been shown to be predictive of the severity of dying-back sensory neuropathy [30].

## Discussion

This study investigated the use of proprioception in the proximal joints of the upper limb to determine if proximal proprioceptive dysfunction exists in cancer survivors who received OX-containing chemotherapy. We implemented three multidirectional sensorimotor tasks: force matching, target reaching, and postural stability tasks to evaluate various aspects of proprioception and its use in simple motor tasks. We found that cancer survivors treated with OX exhibited less accuracy and precision than controls when they had to rely only on proprioceptive information to complete the force matching task. There were also small differences in the postural stability task but no significant differences in the reaching task. The force matching deficits in cancer survivors were significantly correlated with self-reported motor dysfunction. These results suggest that cancer survivors post OX chemotherapy exhibit proximal proprioceptive deficits, and that the deficits in accurate force production are larger than those in kinematic control for the tasks we considered. Force matching tasks similar to those used here could provide a clinically meaningful approach to quantifying OX-related movement dysfunction during and after chemotherapy.

### The most significant deficits in cancer survivors were in the force matching task

Among the three sensorimotor tasks, cancer survivors demonstrated the most significant deficits in force matching. All the sensorimotor tasks we evaluated require sensing proprioceptive signals and generating an appropriate motor response. Force matching required sensing and generating limb forces, whereas the reaching task depended more on perceiving and controlling limb position (kinesthesia). The postural stability task required sensing and controlling force and position. Our results showed the worst deficits in force matching, mild deficits in postural stability, and no deficits in target reaching. This suggests that tasks involving force sensing and generation might be more affected in cancer survivors than those dependent on kinesthesia.

Our cancer survivors demonstrated a larger visual dependency in completing the force matching task compared to controls, indicating deficits in somatosensory feedback. Increased visual dependency has been previously observed in cancer patients with CIPN during standing balance. Many postural studies reported a greater increase in postural sway in the absence of vision in cancer patients than that in healthy controls and attributed such changes to CIPN-related somatosensory deficits [31–33]. However, the type and location of the somatosensory deficits were often overlooked in these postural studies. Kneis et al. [33] suggested involvement of proprioceptive dysfunction but did not specify the location of the deficits; others simply attributed the deficits to abnormal sensory symptoms in distal limbs (e.g., feet) without assessing the proximal joints [31, 32].

Our study is the first to demonstrate the proximal involvement of the somatosensory deficits in cancer survivors. The number of studies considering proximal deficits is very limited. Osumi et al., [34] compared distal and proximal deficits by examining the smoothness of the grasping and reaching performance in the upper limb. They found impaired grasping smoothness and normal reaching smoothness, and concluded that somatosensory deficits occur primarily in the hands [34]. Similar to Osumi et al., [34] we did not find a significant deviation of the reaching performance in our patient cohort from healthy controls. However, we found deficits in force matching ability. This suggests that unique proximal deficits occur in cancer survivors in addition to the distal deficits reported.

### Force matching deficits varied across cancer survivors and directions

The severity of the force matching deficits was not uniform across participants. Some cancer survivors had performance comparable to the healthy controls, others had mild performance deficits, and a subset of them had substantial deficits. This large inter-subject variability likely contributed to the large confidence bounds observed in the accuracy estimates for cancer survivors (Fig. 5). The large inter-subject variability is consistent with different levels of sensory and motor symptom severity reported by our patient cohort (Fig. 7), which was consistent with that reported in the literature [35].

Subjects with the most impaired force matching performance, exhibited the largest errors when generating forces along the axis of the forearm (Fig. 7d). These cancer survivors tended to overshoot the targets when generating force in the directions of 150° and 330°. They also had a higher inter-trial variability for these same directions. There are two biomechanical factors that may have contributed to these results. First, forces generated along the axis of the forearm require only torques about the shoulder, not the elbow, suggesting proximal deficits in force production were responsible for these motor errors. Second, arm strength is not uniform in the horizontal plane used for our measurements. Individuals tend to be strongest when generating forces along the direction of the forearm [36]. Since our task was to generate a constant force of 10 N in all tested directions, the required effort would have been smallest in the directions along which force matching errors were largest. This could suggest that cancer survivors have greater deficits in perceiving and matching forces at lower levels of muscular activation. However, the protocol we used in these experiments was not designed to evaluate this possibility.

### Potential mechanisms contributing to the force matching deficits

Several physiological mechanisms may have contributed to the observed deficits in force matching ability and the closely related sense of effort. The sense of muscle force and effort is thought to arise from two sources of information, a copy of motor commands relayed to the sensory areas (efferent copy) and peripheral signals from sensory receptors, including Golgi tendon organs and muscle spindles [37]. Humans likely use efferent and afferent information to sense muscle force and effort. There is growing evidence that the signals contributing most to the sense of force and effort are task-dependent. For example, Monjo et al. [38] found that muscle spindles contribute to the sense of effort when completing a one-arm force matching task but that central and peripheral sources are important when completing two-arm force matching. In contrast to the perception of effort, the sense of muscular force during one-arm [39] and two-arm [40] matching tasks has been commonly attributed to Golgi tendon organs. Together these studies suggest that signals from peripheral sensory organs were critical for the completion of our force matching task, regardless of whether they followed our instructions to match the target forces or instead relied on the perceived effort to complete the tasks in the absence of vision. Oxaliplatin has been shown to disrupt the signaling function of muscle spindles and Golgi tendon organs [11]. Thus, dysfunction in either could have contributed to our findings.

Deficits in motor output may also have contributed to the performance deficits we observed. Most participants tended to overshoot force targets when visual feedback was absent. This overestimation of force is consistent with previous force matching studies [22, 41–43]. The magnitude of the overestimation for controls and some cancer survivors fell within the reported range of 1-3.5 N. However, a subset of cancer survivors exhibited force errors more than two times the healthy range (Fig. 7a). One possible explanation is that cancer survivors are weaker and more easily fatigued. Thus, matching the 10-N force might be too challenging for them. While no subject reported fatigue or requested extra rest time between trials, we cannot rule out this possibility since weakness and fatigue are common in cancer survivors, and the underlying causes could be multifactorial [44]. Recently, new evidence of OX inducing erratic firing in motor neurons emerged in a preclinical study, and this change in motor neuron function could also contribute to impaired motor control and fatigue in cancer survivors [45]. More complete assessments of strength and motor output variability could help distinguish between these possibilities.

## Limitations

Several limitations of our study need to be addressed. First, our sample size was small. Still, the distribution of symptom severity in our patient cohort was similar to that reported in the literature. A recent study of 144 patients reported that the majority (91%) of the patients receiving OX chemotherapy were asymptomatic or presented with mild functional deficits that were not interfering with activities of daily living [35]. This was also reflected in our study, where all cancer survivors reported mild symptoms and functional deficits, except for one cancer survivor who had no symptoms. Our patient pool did not include patients with more severe functional deficits due to challenges in recruiting this group. Given that the severity of the force matching deficits is correlated with perceived motor dysfunction, we expect cancer survivors with more severe functional deficits to also present with force matching deficits. Second, the sensorimotor tasks we used were designed to emphasize the use of proximal joints (the elbow and shoulder), so as to emphasize the use of proprioception rather than the more distal abnormal sensory symptoms (numbness/tingling, impaired light touch, vibration) commonly attributed to dying back neuropathy. Although none of our subjects reported abnormal sensory symptoms in the proximal arm, detailed neurophysiological assessments such as nerve conduction tests, would need to be conducted to rule out the possibility of any proximal neuropathies. Third, our experimental paradigm did not eliminate cutaneous input from contributing to the force matching deficits, especially in the distal limb where the hand and forearm touched the orthoses. Given that the force deficits were not uniform across directions, the possibility that cutaneous dysfunction is entirely responsible for the observed deficits is unlikely. Lastly, deficits in memory and recall could affect force matching accuracy, but we think that these effects were minimal if present at all. First, if memory or recall was an issue, we would expect considerable deficits in all three tasks, but we only observed significant deficits in force matching. Second, we recorded perceived attention and memory deficits as parts of the C30 questionnaire and found no correlation between the perceived memory deficits and force matching deficits (r = 0.37, *p* = 0.22). Therefore, we believe it is unlikely that memory and recall deficits contributed substantially to performance in our force matching task.

## Conclusions

We implemented three sensorimotor tasks to assess proprioceptive deficits in the proximal joints of cancer survivors treated with OX. The greatest deficits were observed in tasks that required subjects to perceive and generate forces. These results demonstrate that proprioception-related sensorimotor dysfunction occurs in the proximal joints, in addition to previously reported distal deficits. It remains to be seen if cancer survivors also present with purely motor dysfunction that contributes to the observed sensorimotor dysfunction. Currently, clinical assessments of chemotherapy-related movement dysfunction are largely limited to examinations and questionnaires about distal symptoms. Our results suggest that changes in proximal function should also be considered, especially the force matching ability. Such assessments have proved useful in other neurological disorders. For example, the severity of proprioceptive deficits following stroke has been shown to be an important prognostic factor for functional recovery [46]. The ability to accurately match forces has been proposed as a specific target for assessment and intervention within stroke rehabilitation [21]. Although the mechanisms of the injuries between OX chemotherapy and stroke are different, force matching ability might also be an important target of assessment and intervention for OX-related movement dysfunction due to its central role in many functional tasks. With the increasing number of cancer diagnoses and improved survival rate, detecting and minimizing cancer- and treatment-related sensorimotor dysfunction is more important than ever for improving the quality of life of cancer survivors. Monitoring the force matching ability in proximal joints could be a starting point towards a more thorough and objective assessment of movement dysfunction in patients treated with chemotherapy.

**Table 1:** Summary of the demographic, clinical, and behavior of the two participant groups

	Patients	Controls
**Demographic profile**		
Sex [F:M, n]	7:6	8:5
Age* [mean ± std, year]	54 ± 11.6	56 ± 5.1
**Medical profile**		
Height [mean ± std, cm]	171 ± 11.1	167 ± 6.0
Weight [mean ± std, kg]	75.6 ± 9.9	66.8 ± 15.1
BMI [mean ± std, kg/m^2^]	26.0 ± 4.4	24.1 ± 4.6
No. of Charlson comorbidity conditions in addition to cancer diagnosis [mean ± std]	1.2 ± 1.0	–
**Oncological diagnosis**		
Colon cancer [n]	11	–
Rectal cancer [n]	2	–
**Disease stages**		–
I	1	–
II	0	–
III	8	–
IV	4	–
**Chemotherapy**		
Regimen		
Folfox	11	–
Capox	1	–
Folfoxiri	1	–
Total dose [mean ± std (range), mg/m^2^]	763.8 ± 197.7 (1080–470)	–
Time since last treatment [mean ± std (range), months]	7.7 ± 5.6 (0.3–19.9)	–
**Behavioral profile**		
Smoking [yes:no, n]	0:13	0:13
Alcohol [yes:no, n]	9:4	8:5
Weekly intake [mean ± std, alcohol unit]	4.4 ± 5.6	4.6 ± 4.8
Physical activity^◊^ [n]		
Light (activity score ≤ 6)	1	1
Moderate (activity score 7–12)	5	3
Heavy (activity score 13–18)	7	9

*Two-sample t-test of the age showed no significant age difference between cancer survivors and controls (*p* = 0.56)

^◊^Activity score was defined as the sum score of activity type (light: 1; moderate: 2; vigorous: 3) multiplied by the frequency (3–4 times per week: 3; 1–2 times per week: 2; 1–2 times per month: 1; not at all: 0). For example, performing light activity 3–4 times per week and moderate activity 1 to 2 times per week will yield 1 × 3 + 2 × 2 = 7

## Data Availability

The datasets used and/or analyzed during the current study are available from the corresponding author on reasonable request.

## Abbreviations

OX: Oxaliplatin
CIPN: Chemotherapy-induced peripheral neuropathy
EORTC QLQ-CIPN20: European organization for research and treatment of cancer quality of life chemotherapy-induced peripheral neuropathy 20-item quality of life questionnaire
EORTC QLQ-C30: European organization for research and treatment of cancer quality of life core questionnaire
mTNS: Modified total neuropathy score
